# Supervision for the Public Health Services for Older Adults Under the Background of Government Purchasing: An Evolutionary Game Analysis Framework

**DOI:** 10.3389/fpubh.2022.881330

**Published:** 2022-05-16

**Authors:** Canyou Wang, Weifang Cui

**Affiliations:** ^1^School of Humanities, Chang'an University, Xi'an, China; ^2^Shaanxi Provincial Public Science Literacy and Public Policy Research Center, Chang'an University, Xi'an, China

**Keywords:** public health services, older adults, supervision, government purchasing, evolutionary game

## Abstract

As an important measure to involve services for older adults, the government procurement practices have become a key link for public health services. However, the information asymmetry between public health service purchasers and public health service undertakers triggers a supervision dilemma. Based on this background, this study uses the evolutionary game theory to analyze the symbiotic evolution between local governments and public health service institutions under different reward and punishment mechanisms, explore game evolution, strategy adjustment, and influencing factors of different game subjects, and analyze the necessity and appropriate intensity of dynamic rewards and punishment mechanisms. The results show that: under the static condition, the penalty can change the strategies of local governments to a certain extent, but it is still difficult to achieve complete self-discipline management of public health service institutions. If local governments implement a dynamic reward or penalty mechanism in the supervision process of public health services for older adults, the equilibrium between them tends to be evolutionary stable. For three dynamic mechanisms, a dynamic reward mechanism is more conducive to adopting a self-discipline behavior of public health service institutions, which is helpful to realize the supervision of public health services for older adults. Also, there is a positive correlation between the proportion of public health service institutions who adopt a “self-discipline behavior” strategy and the maximum punishment intensity, and a negative correlation with the reward intensity. This study provides theoretical and decision-making references for governments to explore the promotion and implementation of public health services in older adults.

## Introduction

Nowadays, many countries including some developing countries in the world, face a critical challenge with the rapidly aging population (1). For example, according to China's 7th national population census data, the number of older adults (≥60 years of age) has reached 264 million, which accounts for 18.7% of the total population (2). As the aging population increases, the total expenditure on public health will increase dramatically (3). Due to the changing demographic structures, chronic diseases such as diabetes, dementia, and stroke as well as cognitive and physical decline will become increasingly widespread (4). As an important measure to involve services for older adults, the government procurement practices have become a key link for public health services. For instance, by purchasing services for the older adults (≥60 years of age) that suffered from hypertension or diabetes, the Shengzhou government has been provided public health services for urban and rural residents with a standard ¥40 per person per year (5). Hong Kong has launched the “elderly healthcare voucher” scheme (EHCV) since 2009 and issued five $50 (in HKD) vouchers for each older adult aged 70 years or older, to strengthen public health services such as primary nursing for the elderly (4).

However, the development of public health services for older adults faces an important hurdle: there exists an information asymmetry between public health service purchasers (governments) and public health service undertakers (health service institutions) (6). The role of local governments has altered from direct service providers to service purchasers and regulators (principals), and health service institutions have become the service undertakers (agents) of public health services. The outsourcing contractual relationship between them triggers such a dilemma of information asymmetry. On the one hand, according to the contract, local governments would subsidize the health service institutions after a certain period of time (usually based on the number of older adults they serve). But local governments may have information blind spots resulting from the process of the public health services from service undertakers, and face the dilemma of one-to-many supervision. Hence, local governments cannot grasp all kinds of information in the process of the public health services, resulting in the phenomenon of an unqualified government subsidy fraud in some public health service institutions (abbreviated as PHSIs, the same below). On the other hand, the general cultural level of older adults is not high and also lacks awareness of contemporary developments in the country's public health services policy. Therefore, concerning the public health services for older adults under the background of government purchasing, the phenomenon of falsifying data and swindling project funds with false subjects happens frequently. For example, in 2020, 953 PHSIs had been fined a total of ¥4.547 million for illegally falsifying data of Physical Examination (PE) and Contracted Family Doctors Services (CFDS) among adults aged 65 years or older in Gansu province (7). Therefore, how to develop a framework of supervision strategies to avoid the opportunistic behavior of the PHSIs possible, and investigate how different reward and punishment strategies affect the performance of public health services for older adults should be considered and solved at present.

Based on the background of government purchasing, this study will discuss the evolutionary game process of public health services supervision behavior between local governments and PHSIs under different reward and penalty mechanisms. Specifically, we will analyze game evolution, strategy adjustment, and influencing factors of different game subjects, and explore the necessity and appropriate intensity of dynamic rewards and punishment mechanisms, which would provide theoretical and decision-making reference for governments to explore the promotion and implementation of public health services in older adults.

## Literature Review

The current research that relates to the public health services for older adults mainly focuses on the following three aspects. Firstly, some scholars summarize the patterns and practices of public health services for older adults. Ogden et al. (8) presented the clinical preventive services (CPS) for older adults in the US. The CPS program is of great benefit to immunizations, disease, screening, and behavioral counseling interventions. Tang et al. (9) discussed the older adults' utilization of the essential public health services and the factors associated with it. Secondly, some authors focused on a specific public health problem for older people. Kelsey et al. (10) took a prospective cohort study of 765 community-dwelling women and men to examine risk factors for falls among older people. Gerst-Emerson and Jayawardhana (11) used panel data from the Health and Retirement Study to determine whether loneliness was associated with higher health care utilization among older adults in the US. Angel and Mudrazija (12) took the coronavirus crisis as a scenario and provided a brief overview of public support and the financial and health benefits for older adults. Vozikaki et al. (13) used data on 5,129 adults to examine the utilization of preventive health services in relation to social isolation among older adults. Thirdly, some scholars have deepened their understanding of public health services from one or more theoretical perspectives. Based on the “tripartite subject” framework, Yuan et al. (14) discussed the differences in quality and production efficiency between public institutions and outsourced institutions. According to adverse selection and moral hazard in principal-agent theory, Zhang and Xu (6) designed incentive contract models to relieve asymmetric information problems in government procurement of public services. Based on the assessment of the responsiveness of the service theory, Melo et al. (15) assessed the quality of health care of older adults. Chong et al. (4) used a system dynamics analysis to simulate the impact of various enhanced strategies in the elderly healthcare voucher (EHCV) scheme in Hong Kong.

Throughout the existing literature, scholars have put forward many valuable thoughts and opinions on the development status, successful practice, and common dilemma of the public health services for older adults. However, the existing research still has some limits: (1) Most of the literature mainly focuses on the discussion of the concept, connotation, development mode, countermeasures, and mechanisms of the public health services for older adults from a macro perspective which involves the political or economic relationships. (2) Existing studies provide a theoretical basis for analyzing the supervision for the public health services for older adults among different social subjects. However, studies at the micro-level ignore the interaction between two or more heterogeneous entities, especially lack of discussion on the supervision for the public health services for older adults. Although it is recognized that the development of public health services for older adults needs the collaborative support of relevant subjects such as the local governments, public health services institutions, and older adults (16), it is obvious that descriptive studies of qualitative research are in the majority. Quantitative research methods such as mathematical analysis are rarely used to fully explore the behavioral logic of different subjects. Also, the intensity and scope of the behaviors among them are not quantitatively explained.

Evolutionary game theory mainly focuses on the interaction among different players or groups (17). It provides an effective theoretical tool for discussing the strategic choice and evolutionary logic between the subjects of supervision for the public health services for older adults from a microscopic perspective. This theory has been widely applied in such fields as the new energy vehicle industry (17), international cooperative governance of the COVID-19 (18), privacy protection (19), the sharing and disclosure of COVID-19 information (20), and so on. In addition, the thought of evolutionary dynamics is applied to the reward and punishment mechanisms involved in this article. Reward and punishment mechanisms are often used by governments or other social subjects, which usually have a strong incentive and deterrent effect. Sun et al. (21) considered a hybrid enforcement strategy that imposed both positive and negative incentives on participants simultaneously in the collective-risk social dilemma game. They found that a local sanctioning scheme with pure reward is the optimal strategy. Liu et al. (22) introduced corrupt enforcers and violators into the public goods game (PGG) and explored the necessary conditions for the evolution of cooperation in the corrupt background by using the replicator equation approach. Han (23) discussed whether and when participating in and complying with a costly commitment is an evolutionary stable strategy. The results showed that a sufficient budget for rewarding commitment to compliant behavior had achieved better cooperation than the punishment of non-compliant ones. Szolnoki and Perc (24) introduced a rewarding cooperators strategy into the public goods game and showed that adaptive rewarding can overcome the “second-order free-rider” problem. Also, by relaxing the assumption that allowing players to adapt their sanctioning efforts independence on the success of cooperation, Perc and Szolnoki (25) explored that an adaptive punishment mechanism can able to promote the evolution of cooperation in public goods games, and also determined the necessary conditions for promoting public cooperation. Chen et al. (26) explored the role of institutional sanctioning policy called ‘first carrot in promoting cooperation and showed that this sanctioning policy led to a state of full cooperation at lower cost, made a better performance than rewards or penalties alone under a wider range of conditions. Based on the above facts, this study tries to make up for the deficiency of existing studies and discuss the evolutionary game process of the public health services supervision behavior between local governments and PHSIs under different reward and penalty mechanisms with the help of evolutionary game theory. Specially, we analyze the game evolution, strategy adjustment, influencing factors of different game subjects, and explore the necessity and appropriate intensity of government intervention.

## Model Assumptions and Parameter Settings

Given the supervision for the public health services for older adults, this study analyzes the interest conflict and optimal choice between local governments and PHSIs based on the evolutionary game theory, and proposes the following assumptions:

(1) Participants of the game are local governments and PHSIs. Both parties of the game have bounded rationality. The local governments and PHSIs can be regarded as large and infinite groups. Based on this, we can consider employing evolutionary game theory to analyze the interaction mechanism of behavioral strategies (27).

(2) The participant of this game has only two optional strategies. The strategy set adopted by PHSIs is <self-discipline behavior, fraud behavior>. The fraud behaviors of PHSIs include: misreporting the number of public health services, swindling project funds with false subjects, maliciously reducing service quality to cut costs, disclosing private health information about older adults, and so on. These misbehaviors seriously damage the health benefits of older adults and defeat the market order and the fundamental purpose of public health. The strategy set adopted by local governments is <positive supervision, passive supervision>.

(3) Assuming that *x*(0 ≤ *x* ≤ 1) represents the proportion of PHSIs who adopt the “self-discipline behavior” strategy among all PHSIs; then 1 − *x* is the proportion of PHSIs who adopt the “fraud behavior” strategy. Similarly, *y*(0 ≤ *y* ≤ 1) represents the proportion of local governments who choose the “positive supervision” strategy among all local governments, and 1 − *y* is the proportion of local governments who choose the “passive supervision” strategy.

(4) To further build the payoff matrix of this game, it is assumed that if PHSIs adopt a “self-discipline behavior” strategy, *L* represents the expected returns for them. If they choose “fraud behavior,” they can obtain additional benefits Δ*L* (the motivation for PHSIs to fraud). Whatever the strategy, service costs are essential to PHSIs. However, compared to the “fraud behavior” strategy, PHSIs adopting “self-discipline behavior” have higher service and opportunity costs. Therefore, for PHSIs, the service costs of adopting a “fraud behavior” strategy are normalized to zero for simplicity, and the incremental costs of adopting a “self-discipline behavior” strategy are *C*. Similarly, for local governments, if they choose a “positive supervision” strategy, they need to invest more human resources to monitor what PHSIs do, more financial resources to purchase equipment, and so on. Assumed that the incremental effort costs of adopting a “positive supervision” strategy are *I*. If PHSIs adopt a “self-discipline behavior” strategy, local governments gain social benefits *G*_1_, including government image and reputation enhancement, social welfare improvement, and so forth. Otherwise, local governments would lose social benefits *G*_1_.

(5) In order to effectively motivate and strengthen the supervision of PHSIs and ensure the rights of older adults are not infringed, local governments implement the reward and penalty mechanism. If local governments conduct supervision practices actively, they can reward PHSIs with corresponding subsidies according to PHSIs' performance evaluation. If local governments adopt a “passive supervision” strategy, when PHSIs actively implement a strict management mechanism and perform their duties, they can be appropriately rewarded with corresponding subsidies *S*. However, if PHSIs falsify data, swindle project funds with false subjects, or engage in other fraud practices, they will be punished with penalties *P*.

According to the above assumptions, the payoff matrix for both players in the game is shown in Table 1.

**Table 1:** The payoff matrix of both players.

		**Local governments**	
	**Strategy**	**Positive supervision(y)**	**Passive supervision(1 − y)**
PHSIs	Self-discipline behavior(*x*)	*L* + *S* − *C*; *G*_1_ − *S* − *I*	*L* − *C*; *G*_1_
	Fraud behavior(1 − *x*)	*L* − *P* + Δ*L*; *P* − *I* − *G*_1_	*L* + Δ*L*; −*G*_1_

*Public health service institutions (PHSIs)*.

## Evolutionary Game Analysis Between PHSIs and Local Governments

### Evolutionary Equilibrium Analysis

According to the payment matrix of Table 1, the expected earnings of PHSIs when they choose “self-discipline behavior” and “fraud behavior” strategies are *E*_*PY*_ and *E*_*PN*_ respectively, and the average income is ${\bar{E}}_{P}$. Thus, we can obtain the equations to calculate each, as follows:


(1) $$\begin{array}{l} E_{PY}=y\cdot \left(L+S-C\right)+\left(1-y\right)\cdot \left(L-C\right) \end{array}$$

(2) $$\begin{array}{l} E_{PN}=y\cdot \left(L-P+\Delta L\right)+\left(1-y\right)\cdot \left(L+\Delta L\right) \end{array}$$

(3) $$\begin{array}{l} {\bar{E}}_{P}=x\cdot E_{PY}+\left(1-x\right)\cdot E_{PN} \end{array}$$

Then we can calculate the replicator dynamic equation of PHSIs according to Equation (4):


(4) $$\begin{array}{l} F\left(x\right)=\frac{dx}{dt}=x\cdot \left(E_{PY}-{\bar{E}}_{P}\right)=x\left(1-x\right)\left[\left(S+P\right)y-C-\Delta L\right] \end{array}$$

Similarly, the expected earnings when the local government adopts “positive supervision” and “passive supervision” strategies are *E*_*GY*_ and *E*_*GN*_ respectively. The average earning is ${\bar{E}}_{G}$. Thus, we can obtain the equations to calculate each, as follows:


(5) $$\begin{array}{l} E_{GY}=x\cdot \left(G_{1}-S-I\right)+\left(1-x\right)\cdot \left(P-I-G_{1}\right) \end{array}$$

(6) $$\begin{array}{l} E_{GN}=x\cdot \left(G_{1}\right)+\left(1-x\right)\cdot \left(-G_{1}\right) \end{array}$$

(7) $$\begin{array}{l} {\bar{E}}_{G}=y\cdot E_{GY}+\left(1-y\right)\cdot E_{GN} \end{array}$$

Then we can calculate the replicator dynamic equation of the local government according to Equation (8):


(8) $$\begin{array}{l} F\left(y\right)=\frac{dy}{dt}=y\cdot \left(E_{GY}-{\bar{E}}_{G}\right)=y\left(1-y\right)\left[P-I-x\left(S+P\right)\right]\; \end{array}$$

According to Equations (4) and (8), the replicator dynamic equations of this dynamic system I are as follows:


(9) $$\begin{array}{l} \{\begin{array}{c} F\left(x\right)=dx/dt=x\left(1-x\right)\left[\left(S+P\right)y-C-\Delta L\right]\\ F\left(y\right)=dy/dt=y\left(1-y\right)\left[P-I-x\left(S+P\right)\right] \end{array} \end{array}$$

For this dynamic system I, let the replication dynamic equations *F*(*x*) = 0 and *F*(*y*) = 0, we can get four partial equilibrium points of the system: (*x, y*) = (0, 0), (*x, y*) = (1, 0), (*x, y*) = (0, 1), (*x, y*) = (1, 1). In addition, if *P* > *max*(*I, C* + Δ*L* − *S*), (x^*^, y^*^) is also a partial equilibrium point of system I, where: $x^{*}=\frac{P-I}{P+S}$, $y^{*}=\frac{C+\Delta L}{P+S}$.

According to the method proposed by Friedman (28), the stability of partial equilibrium points in an evolutionary system can be obtained by analyzing the local stability of the Jacobian matrix of the system. Therefore, the evolutionary stability state of equilibrium points can be determined by solving the Jacobian matrix of equilibrium points. Then, the Jacobian matrix of the system can be obtained according to Equation (9)


(10) $$\begin{array}{l} J=\left(\begin{array}{ll} \frac{\partial \left(\frac{dx}{dt}\right)}{\partial x} & \frac{\partial \left(\frac{dx}{dt}\right)}{\partial y}\\ \frac{\partial \left(\frac{dy}{dt}\right)}{\partial x} & \frac{\partial \left(\frac{dy}{dt}\right)}{\partial y} \end{array}\right)=\left(\begin{array}{ll} a_{11} & a_{12}\\ a_{21} & a_{22} \end{array}\right) \end{array}$$

where:


(11) $$\begin{array}{l} \{\begin{array}{l} a_{11}=\left(1-2x\right)\left[\left(S+P\right)y-C-\Delta L\right]\\ a_{12}=x\left(1-x\right)\left(S+P\right)\\ a_{21}=-y\left(1-y\right)\left(S+P\right)\\ a_{22}=\left(1-2y\right)\left[T-I-x\left(S+P\right)\right] \end{array} \end{array}$$

If the partial equilibrium point satisfies the two conditions: det*J* = *a*_11_ · *a*_22_ − *a*_12_ · *a*_21_ > 0 and tr *J* = *a*_11_ + *a*_22_ < 0, the partial equilibrium point denotes the evolutionary stability strategy (ESS). According to the local stability analysis method of the Jacobi matrix, the analysis results are as shown in Table 2.

**Table 2:** Analysis results of local stability.

**Equilibrium point**	**det J**	**tr J**
(0, 0)	(−*C* − Δ*L*)(*P* − *I*)	−*C* − Δ*L* + *P* − *I*
(1, 0)	(*C* + Δ*L*)(−*S* − *I*)	*C* + Δ*L* − *S* − *I*
(0, 1)	(*S* + *P* − *C* − Δ*L*)(*I* − *P*)	*S* − *C* − Δ*L* + *I*
(1, 1)	(−*S* − *P* + *C* + Δ*L*)(*I* + *S*)	−*P* + *C* + Δ*L* + *I*
(x^*^, y^*^)	$\frac{\left(C+\Delta L\right)\left(S+I\right)\left(P-I\right)\left(S+P-C-\Delta L\right)}{{\left(S+P\right)}^{2}}$	0

From Table 2, we can conclude that system I contains two kinds of ESS. If *P* < *I*, equilibrium point (0, 0) is the ESS. If *I* < *P* < *C* + Δ*L* − *S*, equilibrium point (0, 1) is the ESS. If *P* > *max*(*I, C* + Δ*L* − *S*), for (x^*^, y^*^), *det J* > 0, *tr J* = 0. (x^*^, y^*^) is a central point of the system I. For the central point (x^*^, y^*^), the Jacobian matrix J^*^ is: $J^{*}=\left(\begin{array}{cc} 0 & \frac{\left(I+S\right)\left(P-I\right)}{P+S}\\ \frac{\left(C+\Delta L\right)\left(C+\Delta L-P-S\right)}{P+S} & 0 \end{array}\right)=\left(\begin{array}{cc} 0 & \tau_{1}\\ \tau_{2} & 0 \end{array}\right)$. The characteristic equation of J^*^ is $J^{0}=\left|\left(\begin{array}{cc} \lambda & 0\\ 0 & \lambda \end{array}\right)-J^{*}\right|$. Let *J*^0^ = 0, we calculate the characteristic roots, $\lambda_{1,2}=\pm \frac{\sqrt{\Delta }}{P+S}$, where Δ = (*C* + Δ*L*)(*I* + *S*)(*C* + Δ*L* − *P* − *S*)(*P* − *I*) < 0. So, λ_1,2_ are the two purely imaginary characteristic roots. According to the conclusion put forward by Taylor and Jonker (29), (x^*^, y^*^) is a central point but does not have asymptotic stability.

We can explain the periodical fluctuation of the system I by referring to the literature (30), the trajectories for replicator dynamics Equation (9) at the central point (x^*^, y^*^) are:


(12) $$\begin{array}{l} \{\begin{array}{l} x=x^{*}+A_{m}cos\left(\omega t+\phi \right)\\ y=y^{*}+A_{m}cos\left(\omega t+\phi \right) \end{array} \end{array}$$

Where $\omega =\sqrt{\left|\tau_{1}\tau_{2}\right|}$, $A_{m}=\sqrt{{\left[x\left(t=0\right)-x^{*}\right]}^{2}+{\left[y\left(t=0\right)-y^{*}\right]}^{2}}$, and


$$\begin{array}{l} \phi =\{\begin{array}{c} \arctan\left(\frac{\left(y\left(t=0\right)-y^{*}\right)}{\left(x\left(t=0\right)-x^{*}\right)}\right)\;if\;\frac{\left(y\left(t=0\right)-y^{*}\right)}{\left(x\left(t=0\right)-x^{*}\right)}>0\\ \pi +\text{arctan}\left(\frac{\left(y\left(t=0\right)-y^{*}\right)}{\left(x\left(t=0\right)-x^{*}\right)}\right)\;if\;\frac{\left(y\left(t=0\right)-y^{*}\right)}{\left(x\left(t=0\right)-x^{*}\right)}\leq 0 \end{array} \end{array}$$

Then, from the Equation (12), we can conclude that the mixed-strategy trajectory of the dynamic system I is a closed-loop curve around the central point (x^*^, y^*^).

According to the above analysis, the penalty value *P* affects the stable equilibrium, but this influence is not absolute. Specifically, if the penalty is not high enough, either (0,0) or (0,1) may become the ESS of the system. But in any case, the complete self-discipline behavior of PHSIs is difficult to ensure. If the penalty is high, the equilibrium point (x^*^, y^*^) does not have asymptotic stability. So, the evolution trajectory between local governments and PHSIs is a closed-loop curve around point (x^*^, y^*^), and the system will not automatically stabilize to this point. (x^*^, y^*^) is not an asymptotic ESS of the system I.

The above discussion shows that in the game between local governments and PHSIs for the public health services for the aged of the system I, the penalty can change the strategies of local governments to a certain extent, but it is still difficult to achieve complete self-discipline management of PHSIs.

### Simulation Analysis

In order to describe the above analysis conclusions, we use the Matlab R2012a software to simulate the evolutionary process of system I.

#### Simulation 1: P < I

**Figure 1:**
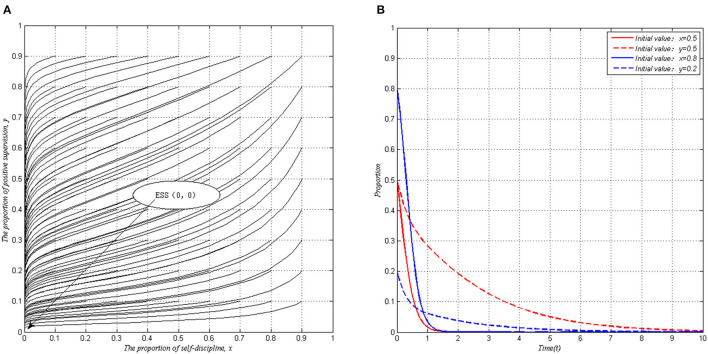
Evolutionary stability of system I when *P* < *I* (*S* = 1, *P* = 1.5, *I* = 2, *C* = 1, Δ*L* = 4). **(A)** Arbitrary proportion. **(B)** Specific proportion.

When local governments adopt a passive supervision strategy, the PHSIs tend to choose a fraud behavior for the revenue of fraud behavior is higher than the revenue of self-discipline behavior. At the same time, when PHSIs do not self-discipline, only rely on a positive supervision strategy of local governments cannot reduce the occurrence of deceptions, and punitive income cannot make up for the positive supervision cost, local governments tend to take a negative supervision strategy. In this condition, the ESS will be <fraud behavior, passive supervision>, as shown in Figure 1.

#### Simulation 2: I <P <C+ΔL − S

**Figure 2:**
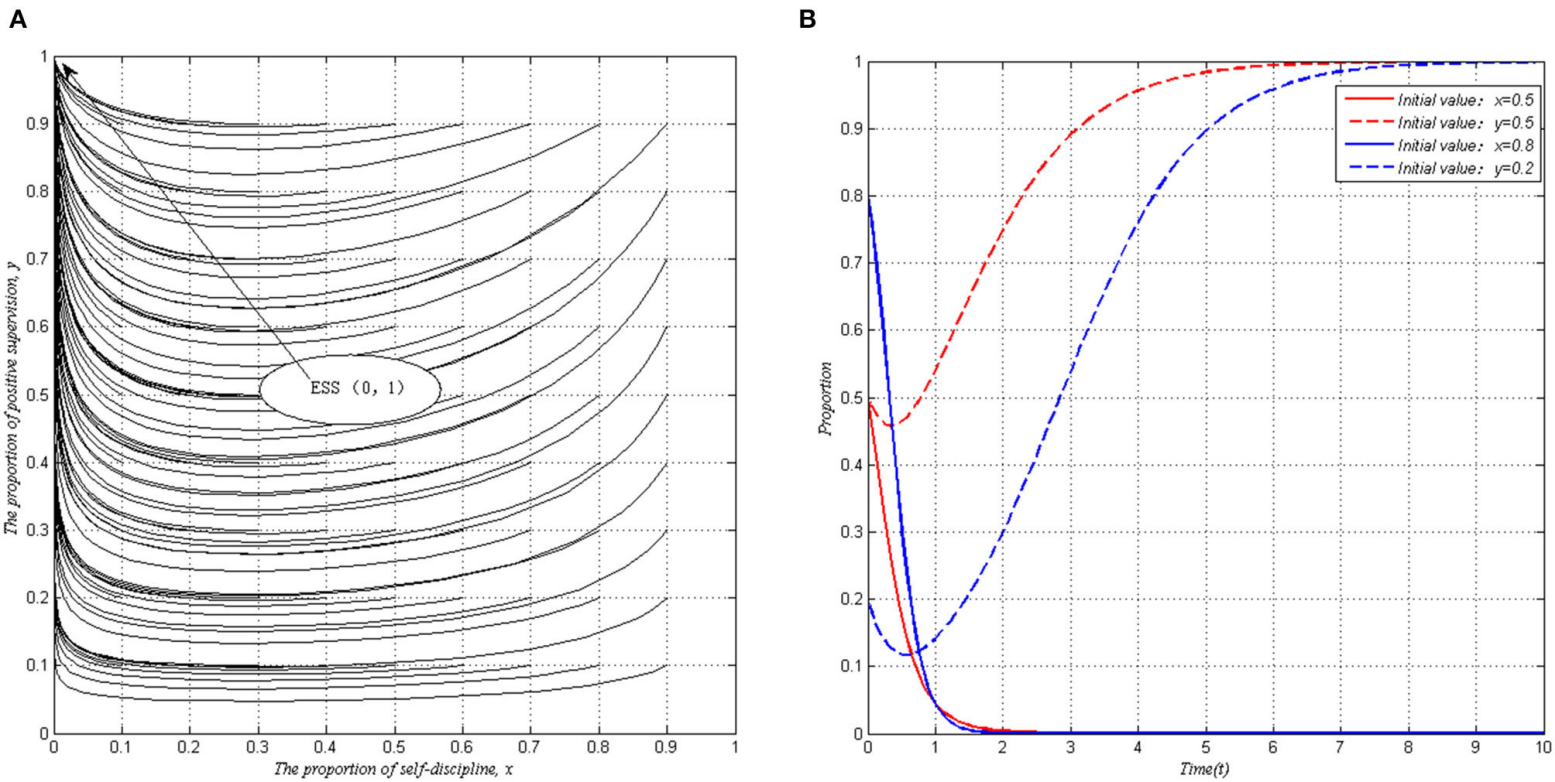
Evolutionary stability of system I when *I* < *P* < *C* + Δ*L* − *S* (*S* = 1, *P* = 3, *I* = 2, *C* = 1, Δ*L* = 4). **(A)** Arbitrary proportion. **(B)** Specific proportion.

This situation is equated such a state with *P* − *I* − *G*_1_ > −*G*_1_ and *L* − *P* + Δ*L* > *L* + *S* − *C*. When PHSIs are not self-disciplined, even though the positive supervision of local governments cannot reduce the occurrence of deceptions, local governments tend to choose the positive supervision strategy, for the punitive income exceeds the cost brought by active supervision. At the same time, if local governments choose a positive supervision strategy, due to *P* < *C* + Δ*L* − *S*, the net income of self-discipline behavior is still lower than that of fraud behavior, and PHSIs tend to choose a fraud behavior strategy. In this condition, the ESS will be <fraud behavior, positive supervision>, as shown in Figure 2.

#### Simulation 3: P >max(I, C + ΔL − S)

**Figure 3:**
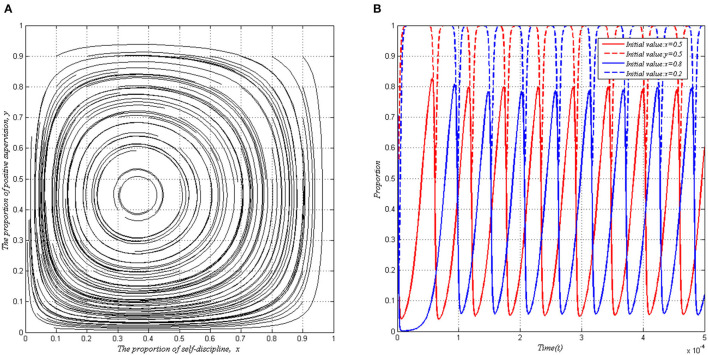
Evolutionary stability of system I when *P* > *max*(*I, C* + Δ*L* − *S*). **(A)** Arbitrary proportion. **(B)** Specific proportion.

In this part, we take a practical case into account in order to illustrate the simulation results of our study. In June 2020, the media reported that one institution offering services for the elderly had gone through the motions in the process of serving the elderly at home. According to the report and survey, this institution served about 8, 000 older adults. The service cost is about ¥105 for each person under the contract. The purchase contract provided by the government stipulates that the service price was ¥150(*S* = 150 × 8000 = 1200, 000). While the real situation was that public health service institutions only spend ¥20–30 yuan to complete the service project(*C* = 105 × 8000 = 840, 000). Therefore, it can obtain additional benefits Δ*L* ≈ 120 × 8000 = 960, 000 (the motivation to fraud). According to the investigation, the government's supervision cost (*I*)for each institution was about ¥10,000. (The government later punished the institution by terminating its contract with the institution and putting it on a bad behavior record list, and asked it for undertaking the liability for breach of contract. This institution could not participate in government procurement practices for the next few years. This shows that the punishment was very severe. And it can be said to be *P* > *ma*x(*I, C* + Δ*L* − *S*) = 600, 000. Figure 3A presents this situation (Suppose *P* = 700, 000). From Figure 3, we can see that the proportion of a self-discipline behavior strategy chosen by PHSIs and the proportion of a positive supervision strategy chosen by local governments move around a fixed central point. The trajectories of the strategies chosen by local governments and PHSIs are closed orbit curves, even if they are near the trajectories of the central point, but they cannot reach it. Although there exists no ESS in the game between PHSIs and local governments, the evolutionary trend of the game indicates that both players are dependent on each other, it is the key care period on which local governments should focus.

The penalty *P* prompts PHSIs to adjust their strategy. If local governments choose a positive supervision strategy, the willingness of PHSIs to choose a self-discipline behavior is improving for fear of bearing a huge punishment cost. However, as time goes by, the local governments face intense pressure to complete numerous supervision activities, so they would gradually adjust their strategy. Once local governments implement a passive supervision strategy, the PHSIs will realize that the revenue of adopting a fraud behavior is higher than the revenue of adopting a self-discipline behavior. When and if that happens, local governments again realize that they tend to choose the positive supervision strategy, for the punitive income exceeds the cost brought by active supervision. Therefore, the probability of PHSIs adopting a self-discipline behavior is constantly fluctuating and this result indicates the game system has no stable state. That is, PHSIs can modify or interact with the strategy selection of local governments.

## Dynamic Reward and Penalty Mechanism

The above section discussed the situation with a fixed penalty and fixed reward, we call this situation “static condition.” Under the static condition, even if the penalty is high enough, the evolutionary game between local governments and PHSIs is periodic, and there is no evolutionary stable strategy. In this section, we will discuss the evolutionary game process of the public health services supervision behavior between local governments and PHSIs under different reward and punishment mechanisms.

### Dynamic Penalty Mechanism

Assume that the penalty intensity of local governments is related to the PHSIs' choice of strategies. Specifically, suppose the penalty imposed by local governments on the PHSIs is proportional to the fraud behavior of PHSIs. That means the higher governments' penalty is associated with the higher proportion of the fraud behavior of PHSIs. So, under the dynamic penalty mechanism, the punishment of PHSIs changes from the fixed value *P* to *P*(*x*) = (1 − *x*)*P*. Then, substituting *P*(*x*) = (1 − *x*)*P* for *P* in the payoff matrix of both players (Table 1), the corresponding replicator dynamic equation of system II is:


(13) $$\begin{array}{l} \{\begin{array}{c} F_{II}\left(x\right)=dx/dt=x\left(1-x\right)\left[\left(P+S\right)y-C-\Delta L-Pxy\right]\\ F_{II}\left(y\right)=dy/dt=y\left(1-y\right)\left[{\left(1-x\right)}^{2}P-xS-I\right] \end{array} \end{array}$$

For the replicator dynamic system II presented by Equation (13), the four equilibrium points are (*x, y*) = (0, 0), (*x, y*) = (1, 0), (*x, y*) = (0, 1), (*x, y*) = (1, 1). If $P>max\left(I,\frac{\left(C+\Delta L\right)\left(C+\Delta L-S\right)}{S+I}\right)$, the point $\left(x_{II}^{*},y_{II}^{*}\right)$ also is another equilibrium point, where $x_{II}^{*}=1-\frac{\sqrt{S^{2}+4P\left(S+I\right)}-S}{2P}$, $y_{II}^{*}=\frac{2\left(C+\Delta L\right)}{S+\sqrt{S^{2}+4P\left(S+I\right)}}$. Similarly, the Jacobian matrix of system II can be obtained according to Equation (13). We can run analysis results of local stability for the four equilibrium points that are (*x, y*) = (0, 0), (*x, y*) = (1, 0), (*x, y*) = (0, 1), (*x, y*) = (1, 1), with a similar way as under static conditions. The results of *det J* and *tr J* for dynamic system II are as follows (see Table 3).

**Table 3:** The results of *det J* and *tr J* for the dynamic system II.

**Equilibrium points**	**det J**	**tr J**
(0, 0)	(−*C* − Δ*L*)(*P* − *I*)	−*C* − Δ*L* + *P* − *I*
(1, 0)	(*C* + Δ*L*)(−*I* − *S*)	*C* + Δ*L* − *I* − *S*
(0, 1)	(*P* + *S* − *C* − Δ*L*)(*I* − *P*)	*S* − *C* − Δ*L* + *I*
(1, 1)	(−*S* + *C* + Δ*L*)(*I* + *S*)	*C* + Δ*L* + *I*

For the equilibrium point $\left(x_{II}^{*},y_{II}^{*}\right)$, the stability of this point is checked by the following calculations.


(14) $$\begin{array}{l} J_{II}^{*}=\left(\begin{array}{ll} a_{11}^{\prime } & a_{12}^{\prime }\\ a_{21}^{\prime } & a_{22}^{\prime } \end{array}\right)=\left(\begin{array}{cc} \frac{\left(C+\Delta L\right)\left[S^{2}+3PS-S\sqrt{S^{2}+4P\left(S+I\right)}+2IP-P\sqrt{S^{2}+4P\left(S+I\right)}\right]}{P\left[S+\sqrt{S^{2}+4P\left(S+I\right)}\right]} & \frac{\left(I+S\right)\left(2P+S-\sqrt{S^{2}+4P\left(S+I\right)}\right)}{2P}\\ \frac{2\left(C+\Delta L\right)\sqrt{S^{2}+4P\left(S+I\right)}\left[2\left(C+\Delta L\right)-S-\sqrt{S^{2}+4P\left(S+I\right)}+2IP-P\right]}{{\left[S+\sqrt{S^{2}+4P\left(S+I\right)}\right]}^{2}} & 0 \end{array}\right) \end{array}$$

Let $\left|\left(\begin{array}{cc} \lambda & 0\\ 0 & \lambda \end{array}\right)-J_{II}^{*}\right|=0$, we calculate the characteristic roots:


$$\begin{array}{l} \lambda_{1,2}^{*}=\frac{\left(C+\Delta L\right)\left[S^{2}+3PS-S\sqrt{S^{2}+4P\left(S+I\right)}+2IP-P\sqrt{S^{2}+4P\left(S+I\right)}\right]\pm \sqrt{\Delta }}{2P\left[S+\sqrt{S^{2}+4P\left(S+I\right)}\right]} \end{array}$$

where $\Delta ={\left[a_{11}^{\prime }\right]}^{2}+4a_{12}^{\prime }a_{21}^{\prime }<0$ and $S^{2}+3PS-S\sqrt{S^{2}+4P\left(S+I\right)}+2IP-P\sqrt{S^{2}+4P\left(S+I\right)}<0$. So, $\lambda_{1,2}^{II}$ is a pair of negative real characteristic roots. In addition, at the equilibrium point $\left(x_{II}^{*},y_{II}^{*}\;\right)$,


(15) $$\begin{array}{l} \{\begin{array}{l} \det J_{II}^{*}=\frac{\left(C+\Delta L\right)\left(I+S\right)\sqrt{S^{2}+4P\left(S+I\right)}\left(-2P-S+\sqrt{S^{2}+4P\left(S+I\right)}\right)\left[2\left(C+\Delta L\right)-S-\sqrt{S^{2}+4P\left(S+I\right)}+2IP-P\right]}{P{\left[S+\sqrt{S^{2}+4P\left(S+I\right)}\right]}^{2}}>0\\ \mathrm{tr}J_{II}^{*}=\frac{\left(C+\Delta L\right)\left[S^{2}+3PS-S\sqrt{S^{2}+4P\left(S+I\right)}+2IP-P\sqrt{S^{2}+4P\left(S+I\right)}\right]}{P\left[S+\sqrt{S^{2}+4P\left(S+I\right)}\right]}<0 \end{array} \end{array}$$

So, $\left(x_{II}^{*},y_{II}^{*}\right)$ is a central point and also has asymptotic stability. It is the ESS of the evolutionary game under a dynamic penalty mechanism.

### Dynamic Reward Mechanism

The main purpose of local governments rewarding the PHSIs is to encourage PHSIs to adopt self-discipline behavior. During the initial stage of the regulation, local governments often provide a higher reward to induce PHSIs to adopt self-discipline behavior. With the proportion of fraud behavior adopted by PHSIs increasing, the willingness of governments regulating in PHSIs decreases. With more and more PHSIs choosing to self-discipline behavior, local governments will gradually reduce the reward level, hence it is the dynamic reward. In other words, the reward of local governments is related to the PHSIs' choice of strategies. Specifically, the higher proportion of fraud behavior of PHSIs is associated with higher rewards to the PHSIs with self-discipline behavior. So, under a dynamic reward mechanism, the reward to PHSIs changes from the fixed value *S* to *S*(*x*) = (1 − *x*)*S*. Then, substituting *S*(*x*) = (1 − *x*)*S* for *S* in the payoff matrix of both players (Table 1), the corresponding replicator dynamic equation of the system III is:


(16) $$\begin{array}{l} \{\begin{array}{c} F_{III}\;(x)=dx/dt=x\left(1-x\right)\left[\left(P+S\right)y-C-\Delta L-Sxy\right]\\ F_{III}\;(y)=dy/dt=y\left(1-y\right)\left[Sx^{2}-\left(P+S\right)x-I+P\right] \end{array} \end{array}$$

For dynamic system III, (*x, y*) = (0, 0), (*x, y*) = (1, 0), (*x, y*) = (0, 1), (*x, y*) = (1, 1) are the four equilibrium points. When $P>max\left(I,C+\Delta L-\frac{SI}{C+\Delta L-S}\right)$, the point $\left(x_{III}^{*},y_{III}^{*}\right)$ also is another equilibrium point, where $x_{III}^{*}=\frac{1}{2}+\frac{P-\sqrt{{\left(P-S\right)}^{2}+4SI}}{2S}$, $y_{III}^{*}=\frac{2\left(C+\Delta L\right)}{P+S+\sqrt{{\left(P-S\right)}^{2}+4SI}}$. Similarly, we can show the results of *det J* and *tr J* for system III as shown in Tables 2, 3. Omitted for brevity.

Similarly, for the equilibrium point $\left(x_{III}^{*},y_{III}^{*}\right)$, let $\left|\left(\begin{array}{cc} \lambda & 0\\ 0 & \lambda \end{array}\right)-J_{III}^{*}\right|=0$, we can calculate the characteristic roots $\lambda_{1,2}^{III}$. Also, we can verify that $\lambda_{1,2}^{III}$ is a pair of negative real characteristicanother roots. In addition, at the equilibrium point $\left(x_{III}^{*},y_{III}^{*}\right)$, the stability of this point is checked by the following calculations.


(17) $$\begin{array}{l} \{\begin{array}{l} \det J_{III}^{*}=\frac{\left(C+\Delta L\right)\sqrt{{\left(P-S\right)}^{2}+4SI}\left[\sqrt{{\left(P-S\right)}^{2}+4SI}-P-S\right]\left[\sqrt{{\left(P-S\right)}^{2}+4SI}-P-S+2\left(C+\Delta L\right)\right]\left[\sqrt{{\left(P-S\right)}^{2}+4SI}-P+S\right]}{4S^{2}\left[P+S+\sqrt{{\left(P-S\right)}^{2}+4SI}\right]}>0\\ \mathrm{tr}J_{III}^{*}=\frac{\left(C+\Delta L\right)}{S\left[P+S+\sqrt{{\left(P-S\right)}^{2}+4SI}\right]}\left[P\left(P-S-\sqrt{{\left(P-S\right)}^{2}+4SI}\right)+2IS\right]<0 \end{array} \end{array}$$

We proceed similarly to the above proof of system III. Also, we can certify that the characteristic roots for the equilibrium point $\left(x_{III}^{*},y_{III}^{*}\right)$ is a pair of negative real, and $\left(x_{III}^{*},y_{III}^{*}\right)$ is a central point and also has asymptotic stability.

### Dynamic Penalty-Reward Mechanism

Assuming that local governments' rewards and penalties to PHSIs are related to PHSIs' discipline behaviors. Substituting *P*(*x*) = (1 − *x*)*P* for *P* and *S*(*x*) = (1 − *x*)*S* for *S* in the payoff matrix of both players (Table 1), the replicator dynamic system IV is:


(18) $$\begin{array}{l} \{\begin{array}{c} F_{IV}\;(x)=dx/dt=x\left(1-x\right)\left[\left(P+S\right)\left(1-x\right)y-C-\Delta L\right]\\ F_{IV}\;(y)=dy/dt=y\left(1-y\right)\left[{\left(P+S\right)x}^{2}-\left(2P+S\right)x-I+P\right] \end{array} \end{array}$$

For dynamic system IV, (*x, y*) = (0, 0), (*x, y*) = (1, 0), (*x, y*) = (0, 1), (*x, y*) = (1, 1) are the four equilibrium points. When $P>max\left(I,\frac{\left(C+\Delta L\right)\left(C+\Delta L-S\right)}{I}-S\right)$, the point $\left(x_{IV}^{*},y_{IV}^{*}\right)$ is also another equilibrium point, where $x_{IV}^{*}=\frac{2P+S-\sqrt{S^{2}+4I\left(P+S\right)}}{2\left(P+S\right)}$, $y_{IV}^{*}=\frac{2\left(C+\Delta L\right)}{S+\sqrt{S^{2}+4I\left(P+S\right)}}$. Similarly, we can show the results of *det J* and *tr J* for system IV as shown in Tables 2, 3. Omitted for brevity.

Also, we can calculate the results of $\det J_{IV}^{*}$ and $\mathrm{tr}J_{IV}^{*}$ for the dynamic system IV. Especially, for the equilibrium point $\left(x_{IV}^{*},y_{IV}^{*}\right)$, we have:


(19) $$\begin{array}{l} \{\begin{array}{l} \det J_{IV}^{*}=\frac{\left(C+\Delta L\right)\sqrt{S^{2}+4I\left(P+S\right)}\left[S+\sqrt{S^{2}+4I\left(P+S\right)}-2\left(C+\Delta L\right)\right]\left[2P+S-\sqrt{S^{2}+4I\left(P+S\right)}\right]}{4{\left(P+S\right)}^{2}}>0\\ \mathrm{tr}J_{IV}^{*}=\frac{\left(C+\Delta L\right)}{2\left(P+S\right)}\left[\sqrt{S^{2}+4I\left(P+S\right)}-2P-S\right]<0 \end{array} \end{array}$$

Finally, we can certify that the characteristic roots for the equilibrium point $\left(x_{III}^{*},y_{III}^{*}\right)$ is a pair of negative real, and $\left(x_{III}^{*},y_{III}^{*}\right)$ is a central point and also has asymptotic stability.

## Numerical Analysis and Discussion

**Figure 4:**
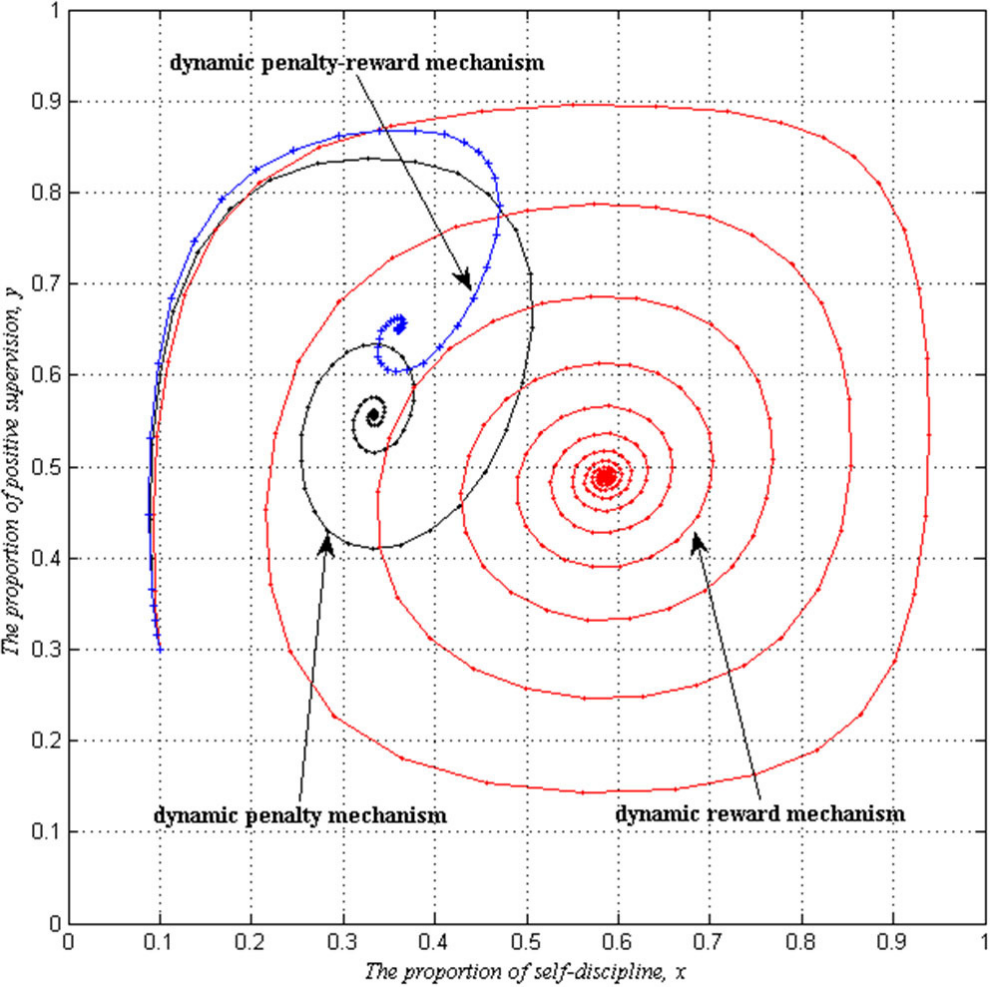
Evolutionary stability of dynamic mechanisms (*S* = 3, *P* = 9, *I* = 3, *C* = 1, Δ*L* = 4).

Comparing the above three kinds of situations, we can find that the effective convergence values of a self-discipline behavior strategy chosen by PHSIs are not the same. Especially, by comparing the values of $x_{II}^{*}$, $x_{III}^{*}$ $x_{IV}^{*}$, it is found that $x_{III}^{*}>x_{IV}^{*}>x_{II}^{*}$. Figure 4 presents this conclusion, and it can be found that the dynamic reward mechanism has the most obvious effect. In this state, the proportion of a self-discipline behavior strategy adopted by PHSIs is the highest, followed by the dynamic penalty -reward mechanism, and dynamic penalty mechanism. Under the dynamic penalty-reward mechanism, local governments are most willing to adopt a positive supervision strategy. If local governments only implement a dynamic penalty mechanism, the convergence degree of the evolutionary game between local governments and PHSIs is the lowest.

In general, although the three scenarios of evolutionary strategies are unable to fully implement PHSIs' self-discipline management behavior, these dynamic mechanisms can make up for the inadequacy of static reward and penalty mechanisms effectively. Under dynamic mechanisms, the evolution of the evolutionary game between local governments and PHSIs tends to a stable focus and the trajectory shows a spiral convergence path.

Next, we analyze the influence of reward and penalty intensity on the evolutionary path of the strategy chosen by local governments and PHSIs. In this sub-section, we take the situation of dynamic reward mechanism as an example to illustrate. Similarly, it can be verified that the other two situations are similar to this situation.

### The Influence of S on the Behavior Strategies Evolution of Both Players

**Figure 5:**
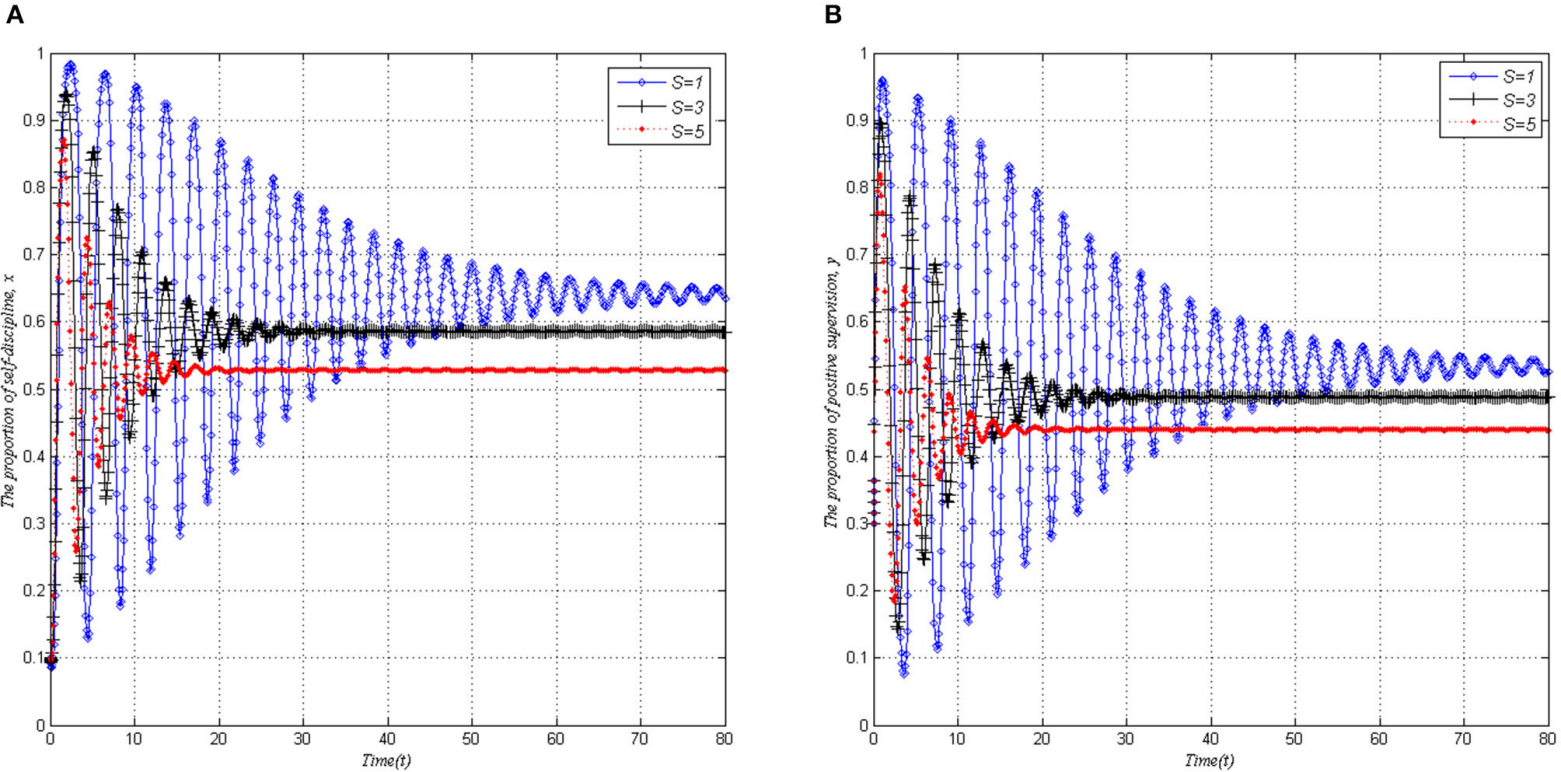
Evolutionary stability of dynamic mechanisms (*P* = 9, *I* = 3, *C* = 1, Δ*L* = 4). **(A)** PHSIs. **(B)** Local governments.

When other parameters remain unchanged, *x* and *y* gradually tend to be stable after experiencing a short-term shock with the increase of the maximum reward *S* of local governments, as shown in Figure 5. As can be seen from Figure 5, the system evolves to a stable state at a faster speed if local governments give a higher reward, but the proportion of the ideal event is both decreased. First, it can be seen from Figure 5B, for local governments, the rewards increase their financial burden, increase the cost of government supervision in a sense, and thus reduce the initiative of active supervision. In addition, the subsidies reduce the self-discipline cost of PHSIs, and local governments would consider that the possibility of implementing the responsibility of self-discipline management by PHSIs increases. Therefore, with the increase of reward intensity, the possibility of local governments' positive supervision behavior decreases.

Second, as shown in Figure 5A, higher rewards weaken the proportion of a self-discipline behavior strategy adopted by PHSIs. This may be contrary to intuition, and the reasons are as follows. On the one hand, this phenomenon can be explained by the snowball effect, that is, when a small snowball starts to move, an external force is needed to expand the motion effect. When it is large enough, the snowball can rely on its inertia to move forward, and the external force is relatively weak. Local governments use higher incentives to promote PHSIs the possibility of implementing the responsibility of self-discipline management strictly. If the size of choosing self-discipline management is large enough, local governments can able to reduce the level of reward and anchor their hope on the inertia of PHSIs' management style. On the other hand, there is no denying the fact that there exists information asymmetry between local governments and PHSIs. Therefore, PHSIs have the moral hazard problem, and such opportunistic behaviors as false reporting and lazy behavior would result in invalid rewards. As Akerlof argued, an excessive subsidy may result in the phenomenon of “bad money drives out good money” (31), which means that not all the rewards are conducive to fully implementing PHSIs' self-discipline management behavior. The rewards reduce the dependence of the chiefs of the fields under their control on the government and force them to actively improve their self-management ability.

### The Influence of P on the Behavior Strategies Evolution of Both Players

**Figure 6:**
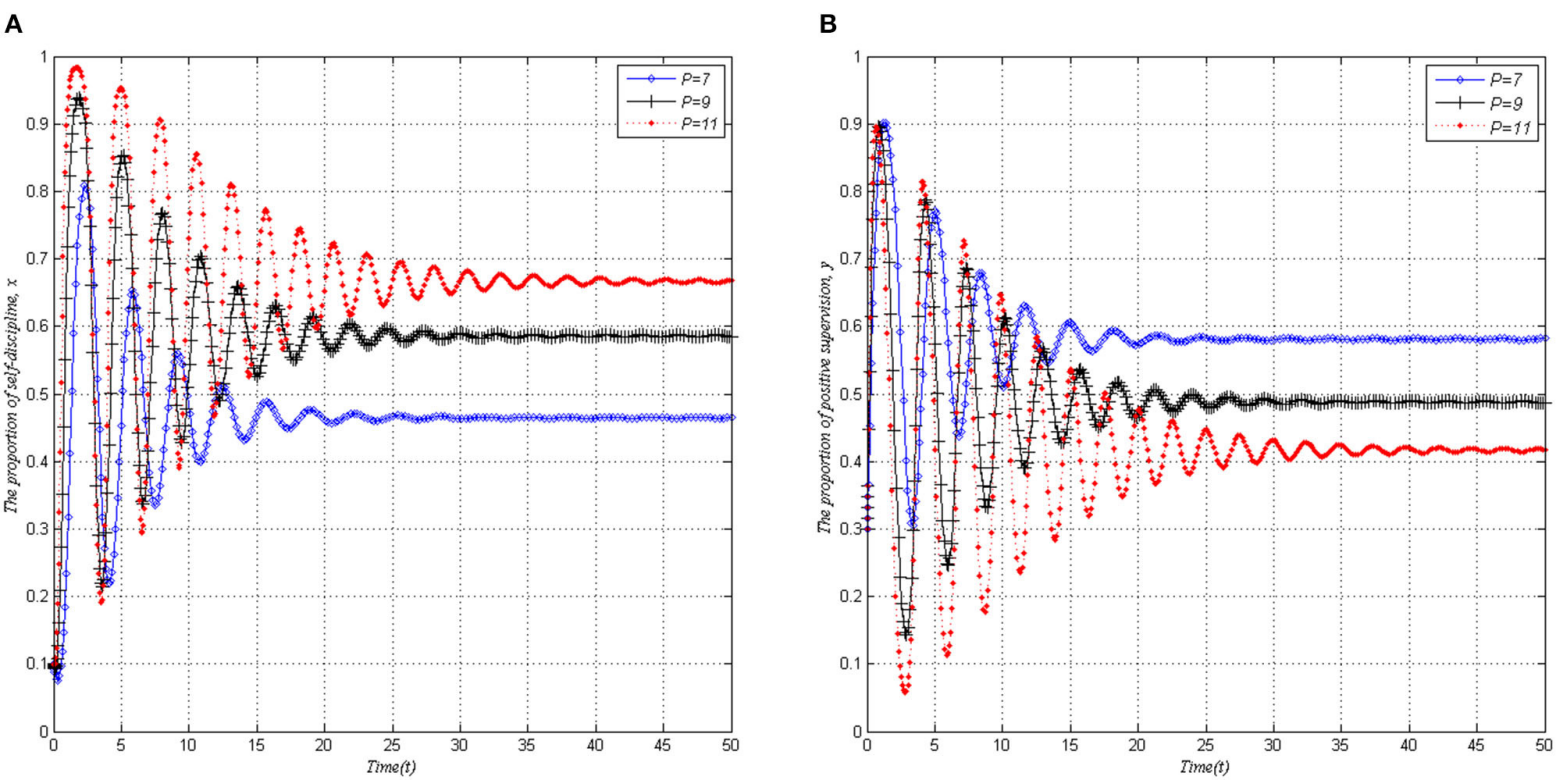
Evolutionary stability of dynamic mechanisms (*S* = 3, *I* = 3, *C* = 1, Δ*L* = 4). **(A)** PHSIs. **(B)** Local governments.

When other parameters remain unchanged, *x* and *y* gradually tend to be stable after experiencing a short-term shock with the increase of the maximum penalty *P* of local governments, as shown in Figure 6. In addition, by comparing Figures 6A,B, we can find that there are two distinctly different trends. As local governments' punishment intensity increases, the proportion of a self-discipline behavior strategy adopted by PHSIs also increases. With the enhancement of local governments' punishment, PHSIs can be urged to implement the responsibility of self-discipline management more strictly, which can further relieve the supervision pressure of local governments, and thus reduce the possibility of their positive supervision.

## Conclusion

Based on evolutionary game theory, this study studies the evolutionary game process of local governments and PHSIs for the supervision of public health services for the aged. The results show that: First, under the static condition, the penalty can change the strategies of local governments to a certain extent, but it is still difficult to achieve complete self-discipline management of PHSIs. Even if the penalty is high enough, the evolutionary game between local governments and PHSIs is periodic, and there is no evolutionary stable strategy between them. Second, if local governments implement a dynamic reward or penalty mechanism in the supervision process of public health services for the aged, the evolutionary strategy between local governments and PHSIs tends to be stable. Third, for three dynamic mechanisms, a dynamic reward mechanism is more conducive to adopting a self-discipline behavior of PHSIs, which is helpful to realize the supervision of public health services for older adults. Lastly, there is a positive correlation between the proportion of PHSIs who adopt the “self-discipline behavior” strategy and the maximum punishment intensity, and a negative correlation with the reward intensity.

## Data Availability Statement

The original contributions presented in the study are included in the article/supplementary material, further inquiries can be directed to the corresponding author.

## Author Contributions

CW conducted the modeling, data analysis, and drafted the original draft. WC revised the manuscript and contributed to the original draft preparation. All authors contributed to the article and approved the final version.

## Funding

This work was partially supported by the National Natural Science Foundation of China (Grant No. 72104036), the Research Planning Foundation in Humanities and Social Sciences of the Ministry of Education of China (Grant No. 21XJC630012), and the Fundamental Research Funds for the Central Universities, CHD (Grant Nos. 300102112607, 300102111606, 300102112501).

## Conflict of Interest

The authors declare that the research was conducted in the absence of any commercial or financial relationships that could be construed as a potential conflict of interest.

## Publisher's Note

All claims expressed in this article are solely those of the authors and do not necessarily represent those of their affiliated organizations, or those of the publisher, the editors and the reviewers. Any product that may be evaluated in this article, or claim that may be made by its manufacturer, is not guaranteed or endorsed by the publisher.
